# Evaluation of the Integrated Epidemiological Surveillance System in Chad according to the CDC method

**DOI:** 10.4102/jphia.v17i1.1762

**Published:** 2026-09-10

**Authors:** Kallah Boukar Ousmane, Moussiliou Noël Paraïso, Harold Dossou Yovo, Oloumane Dyvail Junior, Patrice Zekeng

**Affiliations:** 1 Institut Regionale de Santé Publique Comlan Alfred Quenum (IRSP_CAQ), Université d’Abomey-Calavi, Ouidah, Benin; 2 l’Institut Regionale de Santé Publique Comlan Alfred Quenum (IRSP_CAQ), École des sciences de la vie et de la santé, Université d’Abomey-Calavi, Ouidah, Benin; 3 Africa Synergy Group Plus (ASGPLUS), Cotonou, Benin

**Keywords:** evaluation, performance, Integrated Epidemiological Surveillance System, CDC model, Chad

## Abstract

**Background:**

The Integrated Epidemiological Surveillance System (IESS) is an essential pillar of public health, in the detection and rapid response to priority diseases. In Chad, despite its existence, the persistence of structural weaknesses influences its functioning, which justifies this study.

**Aim:**

This article aims to determine the current performance of the IESS in Chad, with a view to identifying possible shortcomings and proposing corrective measures.

**Setting:**

An evaluative cross-sectional study was conducted from 16 September 2024 to 15 January 2025, in eight provincial health delegations in Chad.

**Methods:**

The collected primary and secondary data were analysed according to the five pillars of the Centres for Disease Control and Prevention (CDC) evaluation methodology: operation, usefulness, importance, cost and quality of the IESS.

**Results:**

The quality of IESS in Chad – evaluated by the CDC method – is considered low with an overall score of 44% (other pillars have been evaluated otherwise). The acceptability (87%) and simplicity (72%) of this system are its strengths. Although acceptable, the flexibility (56%) and responsiveness (55%) of the system need to be improved. The weak points that justify this low quality overall are its low sensitivity (29%) and positive predictive value (29%), as well as its very low representativeness (27%). This overall assessment seems obvious given the weaknesses identified in terms of operation, usefulness, importance and cost of IESS in Chad.

**Conclusion:**

The IESS in Chad remains generally functional but is of low quality.

**Contribution:**

This study innovates mainly because of its rigorous application of the five pillars of the CDC method for assessing the current performance of Chad’s IESS in 2024.

## Introduction

Global life expectancy fell by 1.8 years between 2019 and 2021 as a direct result of coronavirus disease 2019 (COVID-19). Nearly five years after this health crisis, health systems in general and surveillance systems in particular, remain weakened, resulting in delays in vaccination and detection of pathogens.^1^ This is more glaring in Africa, which is experiencing an intensification of health crises, revealing critical gaps in epidemiological surveillance (ES). Indeed, between 2024 and 2025, 125 epidemics were identified on the continent, including 39 high-level health threats and 78 moderate ones.^2^ Since January 2025, 10 944 cases of mpox have been confirmed and 63 deaths have been recorded.^3^ In the Democratic Republic of the Congo (DRC), Ethiopia, Kenya, Nigeria and Uganda, measles outbreaks have been reported, with thousands of cases and high child mortality.^4^ Regarding malaria, Africa Centres for Disease Control and Prevention (CDC), in its latest report in 2025, mentions a shortage of medicines in Namibia and vaccine scepticism in Uganda. These data clearly demonstrate an increase in simultaneous, often cross-border, outbreaks and a failure in early detection, rapid response and risk communication. Various sources generally agree on four explanations for these facts: firstly, low vaccination coverage in rural areas and refugee camps; secondly, delays in the detection and reporting of cases; thirdly, insufficient coordination between national and regional health authorities; and fourthly, the weak integration of the Epidemiological Surveillance System (ESS).

In 2023, a review was commissioned by World Health Organization (WHO) Africa in Chad^5^ to identify significant gaps in the ES despite its existence in Chad’s health system. These are the quantitative and qualitative inadequacy of medical infrastructure, the understaffing of qualified health personnel, inaccessible areas because of geographical constraints or the hostile security context, the insufficient funding of several programmes and the verticalisation of programmes with insufficient pooling of the few available resources.^6^ Yet, it is scientifically established that countries with underperforming emerging markets (EMs) or those without a community-based ES are not able to detect and respond to public health threats or events in a timely manner.^7^ In addition, the quality of an ESS is essential for public health, as it illustrates the ability of a country or region to effectively detect, analyse and respond to health threats.^8^ Therefore, it becomes more than urgent to assess the quality of the ESS to identify dysfunctions and propose solutions that can improve its performance in the entire ES process. It is with this in mind that we conducted this study, which aims to evaluate the current performance of the Integrated Epidemiological Surveillance System (IESS) in Chad according to the CDC method.

## Research methodology and design

### Scope of the study

With an area of 1 284 000 km^2^, Chad is ranked as the fifth largest country in Africa. According to INSEED-Chad (2020), the Chadian population is estimated at about 16 244 513 inhabitants, that is, a density of 12.5 inhabitants per square kilometre, well below the continental average (49.4 inhabitants per km^2^). The country shares its borders with Sudan to the east, Libya to the north, Cameroon, Nigeria and Niger to the west, and the Central African Republic to the south.^9^ The Chadian health network has a three-level pyramidal organisation to ensure progressive health coverage that is accessible to the entire territory: central, intermediate and peripheral. It is recognised that all levels of the health system are involved in the implementation of surveillance activities to detect and control priority diseases and conditions.

### Study type, target population, inclusion-exclusion criteria

This is an evaluative cross-sectional study. The target population is composed of all Integrated Epidemiological System (IES) health workers. Inclusion criteria in this study were any health worker who has resided in the survey area for at least 12 months, works within the IESS, regardless of their profile or position, and has voluntarily consented to the survey. Exclusion criteria were any health worker who has lived at the survey site for less than 12 months, or who does not work in the IESS or was unavailable during the survey period.

### Sampling

The provincial health delegations (PHD) were chosen by simple random draw without discount, which led to the selection of 8 PHD (including Ndjamena by default because of its special status) out of the country’s 23 PHD. In each PHD, a purposive choice of 3 Health District (HD) was made, and 3 Health Centre Managers (HCM) were chosen in each HD, including 1 urban and 2 IES Focal Points (FP). In the specific case of the PHD of Ndjamena, all 5 health HDs and 2 medical practices and/or clinics or care practices were also selected because of the high number of private health structures (PHS), specifically 1 medical or clinical practice and 1 care practice per district. The heads of the IES branch of the PHD directorate and 2 health professionals from the IES directorate, including a data technician, were systematically retained to have better access to information. The final sample size resulting from this procedure, is 150 agents.

### Atlanta CDC evaluation model

Among the models for evaluating epidemiological surveillance systems, the one proposed by the Atlanta CDC remains the best known. The same group of authors has published it many times in various scientific journals with a large international audience. This model is used in several epidemiology teaching textbooks, which are widely disseminated and used. It is also a reference in this field. This is why we use this model, which includes various dimensions of analysis whose relative importance must be weighted according to the surveillance system studied.^10^ This evaluation model is based on the analysis of following five main criteria:

- **Description of how the IESS works:** At this level, it will be a question of listing and describing the distinct stages of the surveillance process as recommended by the WHO, while contextualising each stage for the IESS in Chad. The synthesis of these contextualised activities will be represented on a functional diagram.^10^
- **Usefulness of the IESS:** Because the IESS in Chad does not duplicate other sources of information, the answers to the eight utility evaluation questions, according to Dabis et al., (see Table 1-A3 in Appendix 3) will give an idea of its usefulness.^10^
- **Importance of the IESS:** It will be assessed by the number of cases, the lethality and morbidity of the diseases under surveillance in Chad. Even rare or perceived minor events can be crucial if they signal an emerging risk or a possible re-emergence of controlled diseases.^10^
- **Evaluation of the costs of the IESS:** Given the opacity that characterises the financial management of the IESS in Chad, we did not have access to data on the direct and indirect costs related to the operation of the IESS. As a result, as part of this study, we assessed human resource needs, equipment and logistics needs, and financial needs.
- **Quality assessment of a surveillance system:** The evaluation of quality according to the CDC model considers eight indicators, namely simplicity, flexibility, acceptability, sensitivity, specificity, Positive Predictive Value (PPV), representativeness and responsiveness. In this study, we did not consider predictability because of a lack of available evidence. For each indicator selected, we considered the definition of Dabis et al.^10^ which we contextualised to the realities of Chad. Appendix 1 presents the various criteria identified for the quantification of the selected indicators, as well as the sources of the data and the expected scores.

### Data collection

This study used both primary and secondary data. Primary data represent information collected from IESS staff in Chad in the sample constituted, as part of a field survey. These data were supplemented by secondary data, from the detailed reading and analysis of working articles (action plans, activity reports, technical tools and various databases), theses and books on the subject, as well as routine data from the institutions in charge of IESS in Chad. Three data collection tools were used: a standardised questionnaire (digitised using Kobo Toolbox), an observation grid and a follow-up sheet for the investigated cases.

### Data processing and analysis

The clearance consisted of not only correcting errors and inconsistencies that crept in during the collection, removing duplicates, to label the various variables, but also creating composite variables or break others down as required.

As the variables are essentially categorical, we have summarised them using their numbers and proportions. For data analysis using the CDC method, we produced global or regional frequency tables, as well as graphs adapted to the nature of the variables and the purpose of the analysis. Thus, depending on the nature of the variables used, we designed pie charts, bar charts and radars (the case of the quality of the IESS).

For the specific case of the IESS quality assessment, the data analysis consisted of the estimation of the scores: the scores of the criteria, the scores of the indicators and the overall score.

#### Criteria score

Three estimators were used to calculate the scores for the criteria: proportion, rate and dummy. Proportion and rate were calculated using primary data, while dummy was assessed using secondary data. The estimator of the proportion *p* is defined as follows (Equation 1):


$$p=\frac{Number\;of\;agents\;verifiying\;criteria}{Total\;Number}\times 100$$ [Eqn 1]

The total number represents the size of the sample in the case where the criterion concerns all respondents. The rate estimator *t* looks as follows (Equation 2):


$$t=\frac{Number\;of\;event\;occurrence}{Exposed\;population}\times 100$$ [Eqn 2]

The exposed population represents the sample size if the event is reported by all respondents. The dummy *d* estimator of criterion *i* is defined as follows (Equation 3):


$$d_{i}=\{\begin{array}{c} 1\;if\;criteria\;i\;is\;observed\\ 0\;otherwise \end{array}$$ [Eqn 3]

The binary values (*n* = 1/0) of a dummy have been converted into a percentage according to the number of criteria in the indicator (*n* = 1/8 or 13% if the indicator is composed of eight dummy criteria).

#### Indicator score

The score of the indicator is the aggregation of the scores of the criteria it composes. Except for indicators composed of dummy-type criteria, the scores represent the arithmetic mean of the criteria scores. The score of a dummy indicator represents the sum of the proportions (converted binary values) of the criteria it composes. The score of an indicator is between 0 and 1 (between 0% and 100%).

#### Overall score

The overall score is the aggregation of indicator scores. It represents the weighted average of the observed scores for all the indicators evaluated. Taking (between 0 and 1) as the weight of indicator *i* and the observed score of indicator *i*, the overall score is defined by (Equation 4):


$$SG=\sum_{i=1}^{7}s_{i}\times p_{i}$$ [Eqn 4]

The review of the literature consolidated by the opinion of experts in the field allowed us to consider the following weights: simplicity (10%), flexibility (10%), acceptability (10%), sensitivity (25%), PPV (25%), representativeness (5%) and responsiveness (15%).

The assessment of the indicator scores and the overall score was carried out using the Varkevisser scale.^11^ In this case, each indicator is evaluated according to four levels of assessment: Insufficient (if the score is less than or equal to 24%), Low (if the score is between 25% and 49%), Acceptable (if the observed score is between 50% and 74%), Good (if the observed score is greater than or equal to 75%). Excel and STATA 17 software were used for data processing and analysis.

### Ethical considerations

This study examined compliance with research ethics. Indeed, as part of this survey, we obtained authorisation from the Ministry of Public Health and Population of Chad on 26 April 2024, under reference number: 2185/MSPP/SG/2024 of 26 April 2024. An information sheet explaining the study, the freedom to participate and the confidentiality of the information collected was presented to respondents before each interview.

## Presentation and analysis of results

### Sample description

At the end of the data collection, 136 agents out of 150 planned (participation rate of 91%) were interviewed. The distribution by profile shows that the majority (60% [*n* = 82]) were clinicians (9 per province, except N’Djamena [*n* = 19]), followed by district surveillance focal points (32% [*n* = 44]), then Head of the Epidemiological Surveillance Unit (HESU) (a total of 8 or 1 HESU per province), and technicians at the central level were the least represented (*n* = 2). Most respondents (86% [*n* = 117]) were men, compared to a minority of women (14% [*n* = 19]). In terms of educational attainment, just over four in five respondents (82% [*n* = 112]) had a secondary level, 17% (*n* = 23) had a higher education level and only 1% (*n* = 1) reported having a primary level education. As regards their professional situation, most of the staff interviewed were civil servants (78% [*n* = 106]), followed by Ministry of Public Health and Prevention (MPHP) contract workers (18% [*n* = 24]) and community contract workers, who were poorly represented (4% [*n* = 6]). Most of them (88% [*n* = 119]) worked in the public sector, compared to 8% (*n* = 11) in the private sector and 4% (*n* = 6) in the parastatal. In terms of seniority, almost half (48% [*n* = 65]) had between 1 year and 4 years of experience, compared to 39% (*n* = 53) who had 5 years of experience or more. Only 13% (*n* = 18) of the agents surveyed had less than 1 year of experience (see Appendix 2).

### Description of the functioning of the Integrated Epidemiological Surveillance System in Chad

Epidemiological surveillance in Chad is said to be integrated. Twenty-one priority diseases and health events are under surveillance: Meningitis, measles, cholera, acute flaccid paralysis (AFP), neonatal tetanus (NNT), yellow fever, malaria, malnutrition, maternal deaths, Ebola virus disease, hepatitis E, scorpion sting, guinea worm, avian influenza, snake bite, chikungunya, COVID-19, dog bite, diphtheria, dengue and mpox. They include diseases with epidemic potential, diseases selected for eradication or elimination, and other diseases and events of public health significance. Integrated epidemiological surveillance activities are implemented at the operational level by different actors: health workers in health facilities, community workers, focal points in charge of district epidemiological surveillance and the head of the integrated epidemiological surveillance unit. The main activities carried out are:

- **Case identification:** In the case of the CHAD IESS, it should be observed that standardised case definitions do not concern all diseases and are not available in all health facilities.
- **Notification or reporting of cases:** The reporting of cases by the IESS of Chad is in line with the WHO recommendations in this area.
- **Data analysis and interpretation:** In the case of the IESS in Chad, this step is most often carried out at the central level.
- **Investigation and confirmation of cases and possible epidemic outbreaks:** Biological samples received from the peripheral and intermediate levels are generally deposited at the University Hospital Center of National Reference (Centre Hospitalier Universitaire de Référence Nationale [CHURN]) or at the mobile laboratory level at the Chad-China Friendship Hospital depending on the case of the latter. Acute flaccid paralysis samples are deposited directly with the WHO before being sent abroad (either to the Centre Pasteur in Yaoundé in Cameroon or to the Institut Pasteur in Dakar in Senegal) for biological analysis.
- **Preparation:** In the case of the IESS of Chad, kits are lacking in some health facilities. where the kits are prepared, this is not the case for all diseases under surveillance (only for cholera). In addition, capacity building of response responders is needed at all levels of the health system in the event of an outbreak. The organisation of these trainings generally takes time (between 1 week and 2 weeks); which delays the implementation of the response.
- **Response:** Contrary to WHO recommendations for response, the mobilisation of resources, especially material and financial, is slow to occur in the event of an epidemic outbreak in Chad. This explains the lack of coordination of response actions and the inadaptability of these actions to the reality of the epidemic. As a result, despite the resources involved, the fatality rate remains high.
- **Feedback:** In the case of the IESS of Chad, feedback remains very low and deserves to be reinforced with stakeholders, especially for diseases with epidemic potential to anticipate actions to be taken.
- **Evaluation and improvement of the system:** Unfortunately, few evaluations on integrated epidemiological surveillance have been conducted in Chad since the establishment of this surveillance mechanism in 2001. Two external reviews on this integrated epidemiological surveillance are documented, namely: an external review in 2011 and the one in 2024 led by the WHO in collaboration with the MPHP of Chad.

Figure 1-A3 in Appendix 3 presents the summary of the main activities carried out by the IESS in Chad on a functional map.

### Usefulness of Integrated Epidemiological Surveillance System in Chad

Table 1-A3 in Appendix 3 presents the answers to the eight utility evaluation questions according to Dabis et al.^10^ In view of these responses, Chad’s IESS is very useful. Indeed, it has made it possible to detect, monitor and respond to cases of diseases under surveillance, particularly epidemic situations between 2022 and 2024, for example, the common measles epidemic, circulating polio, hepatitis E, dengue fever and diphtheria (Questions 1, 2, 3 and 5). In addition, the data provided by this system enabled the launch of the WHO mission entitled: ‘External Review of Polio and Other Vaccine-Preventable Diseases Surveillance in Chad, 2024’ (Questions 4 and 8). However, this usefulness is confronted with financial opacity and a lack of evaluation. Because of opacity in managing the system’s financial resources, we cannot assess its contribution to the planning and allocation of resources to a programme (Question 6). Similarly, the ESS of Chad does not yet have an impact assessment report on measures to prevent or control health problems (Question 7).

### Importance of Integrated Epidemiological Surveillance System in Chad

Table 2-A3 in Appendix 3 provides an overview of the evolution of several diseases and health events between 2021 and 2024. It also highlights epidemiological trends, fluctuations in the number of cases and deaths, and the emergence or disappearance of certain pathologies. The analysis of these data reveals contrasting dynamics depending on the disease, with epidemic peaks or significant decreases in cases for some but also warning signs for others. Indeed, more than 10 000 cases of measles were reported in 2023 followed by a decrease in 2024, but paradoxically an increase in deaths. This lag suggests late detection, incomplete vaccination coverage and an ineffective health response. In addition, the sudden reappearance of diphtheria in 2024, with 2595 cases and 70 deaths, reflects flaws in prevention, and above all a lack of early warning. Also, despite a slight decrease in deaths, more than one million annual cases of malaria continue to saturate the health system. Without close monitoring, control efforts risk running out of steam. Thousands of cases of envenomation (scorpions, snakes) each year have been recorded, yet with a mortality that could be avoided if emergency care was better anticipated and distributed. In maternal and child health, maternal deaths and NNT cases are stagnating at alarming levels, revealing structural deficits in obstetric and neonatal care. Similarly, malnutrition is on the rise, reflecting socio-economic vulnerabilities that only targeted surveillance can illuminate and correct. Finally, the emergence of emerging diseases such as mpox and dengue fever in 2024 shows that the epidemiological landscape is changing rapidly, and that inaction is costly. In this context, where some diseases are under control (cholera, Ebola, guinea worm), but where others emerge or persist, vigilance must never weaken. Strengthening the surveillance and effectiveness of the IESS in Chad means protecting lives, anticipating crises, and building a resilient health system.

### Cost of running the Integrated Epidemiological Surveillance System in Chad

This component responds to the need for human, material, logistical and financial resources to be deployed by the Ministry and its partners for the implementation of IESS activities in Chad.

#### Human resources requirements

The results of the human resource estimates are presented in Table 1-A4 of Appendix 4. Overall, 105 officers or 77% expressed the need for additional staff for monitoring activities. In the Lake region, all the participants (i.e. 100% [*n* = 16/16]) are unanimous on this subject. The analysis of Figure 1-A4 of Appendix 4 shows that 79% (*n* = 81/105) of the profiles sought are nurses and/or State-Registered Nurse (STN) as support staff for surveillance activities. In addition, almost half (49% or *n* = 69) of the officers surveyed said they had not received any training on EM. There are strong regional disparities, with regions clearly lagging in terms of training, particularly in Borkou (85% [*n* = 11/13]) and Logone Occidental (71% [*n* = 10/14]). Although all SOCs and technicians at the central level have been trained, some district focal points (20% [*n* = 9/44]) and most clinicians (71% [*n* = 58/82]) have not been trained (Figure 2-A4 and Figure 3-A4 Appendix 4).

#### Equipment and logistical resource requirements

Table 2-A4 of Appendix 4 presents the requirements expressed in terms of material and logistical resources. Overall, 12% (*n* = 16/136) reported being out of stock of tools and collection kits. Although collection kits seem to be available, they are not always complete. In fact, 32% (*n* = 32/119) of the staff have incomplete kits (Figure 4-A4 in Appendix 4).

Unlike other resources, means of travel are scarce for surveillance in Chad. According to the results of the survey, overall, 93% (*n* = 126/136) of respondents do not have a means of transport. At the provincial level, some provinces do not have any at all, such as Borkou and Chari Baguirmi, Mayo Kebbi East and Salamat. The other regions have few (20% or less). No branch chief, let alone DSEI technicians, have a functional means of transport and barely 9% (*n* = 4/44) of the focal points have it (Figure 5-A4 in Appendix 4).

Overall, 7% (*n* = 10/136) of the officers surveyed stated that communication tools for the transmission of surveillance data were not available. The province of Salamat expresses the highest need with 18% (*n* = 3/16) of reported unavailability, followed by Logone Occidental (14% [*n* = 2/14]), then Lake (6% ([*n* = 1/16]) and Mayo Kebbi (14% [*n* = 7/16]).

Regarding the availability of the cold chain, a quarter of the respondents (25% [*n* = 4/16]) in the Lake region say they do not have one, followed by Logone Occidental with 14% (*n* = 2/14), Chari Baguirmi with 13% (*n* = 2/16) and N’Djamena with 7% (*n* = 2/30). Among respondents who reported having a cold chain, the functionality rate is 100% (*n* = 128/128).

#### Financial resource requirements

It is difficult to establish the total cost allocated to integrated epidemiological surveillance activities because the technical directorate of surveillance, the public health emergency operations centre, the management of the expanded programme on immunisation and the PHDs of Chad do not communicate or share information on the financial aspects despite our various requests. Nevertheless, our investigations allowed us to obtain the amount allocated to epidemiological surveillance at the level of the MPHP through the Directorate of Finance and Budget (Table 3-A4 in Appendix 4).

Indeed, the budget allocated to monitoring has not reached 1% of the overall budget of the MPHP over the last 3 years. Already insignificant, this proportion decreased over the period. This result illustrates the crying and urgent need of the Integrated Epidemiological Surveillance System (IESS) in Chad in terms of financing its activities. The analysis of Figure 5-A4 in Appendix 4 shows that almost all of the staff members surveyed (93% [*n* = 50/54]) reported arrears in the payment of their incentive bonus for supervisory activities. The number of months in arrears varies from officer to officer, as shown in Table 3-A4 of Appendix 4. Indeed, just over half (52% [*n* = 28/54]) of the Supervisory Focal Points reported fuel premium arrears of more than 1 year and 41% (*n* = 22/54) (1–6 months and 7–12 months) of fuel premium arrears of less than 1 year. Only 7% (*n* = 4/54) are up to date in the payment of fuel premiums.

### Assessment of the quality of Integrated Epidemiological Surveillance System in Chad

#### Simplicity of Integrated Epidemiological Surveillance System in Chad

Table 1 presents the observed scores for the simplicity criteria. The simplicity of the IESS in Chad is considered acceptable, with an observed score of 72% of the expected score. This simplicity can be explained by the relatively high time taken to confirm prices (99% [*n* = 135/136]), the existence of the case definition (93% [*n* = 126/136]) and its mastery by the surveillance officers (98% [*n* = 133/136]). However, the relative complexity of this system stems from the mandatory nature of case confirmation (12% [*n* = 17/136]).

**TABLE 1:** Assessment of the simplicity of the Integrated Epidemiological Surveillance System (*N* = 136).

Simplicity criteria of the IESS	Expected score (%)	Observed score	
		*n*	%
Time to confirmation of cases	100	135	99
Existence of the case definition	100	126	93
Knowledge of the case definition	100	133	98
Mandatory laboratory confirmation of cases	100	17	12
Dependence on associated services	100	76	57

**Average score**	**100**	**-**	**72**

Note: Assessment (Varkevisser) = Acceptable.

IESS, Integrated Epidemiological Surveillance System.

#### Flexibility of Integrated Epidemiological Surveillance System in Chad

Table 2 presents the observed scores of the flexibility criteria. With a final observed score of 39% of the expected score, the flexibility of the IESS in Chad is considered acceptable. This assessment is explained by the fact that the system faced difficulties in adapting during the COVID-19 crisis, related to the lack of adequate equipment, the insufficient funding required and the health incivility of the community, which did not apply the IESS’s recommendations on containment and hygiene during the health crisis.

**TABLE 2:** Assessment of the flexibility of the Integrated Epidemiological Surveillance System.

Flexibility criteria for the IESS	Expected score (%)	Observed score (*n* = 8)	
		*n*	%
Simplicity-driven flexibility	13	1	13
Weaknesses found during the last epidemic	13	0	0
Low health coverage	13	0	0
Limited emergency response capacity	13	0	0
Shortage of medical resources	13	0	0
Coordination and weak governance	13	1	13
Non-responsive information system	13	1	13
Health incivility at the community level	13	0	0

**Total score**	**100**	**-**	**39**

Note: Assessment (Varkevisser) = Low.

IESS, Integrated Epidemiological Surveillance System.

#### Acceptability of Integrated Epidemiological Surveillance System in Chad

Table 3 presents the observed scores of the acceptability criteria. The acceptability of IESS in Chad is considered good, with an observed score of 87% of the expected score. This evaluation illustrates the strong commitment of the supervisory system staff, the timeliness of file processing and the completeness of forms and reports.

**TABLE 3:** Assessment of the acceptability of the Integrated Epidemiological Surveillance System.

Criteria for acceptability of the IESS	Expected score	Observed score		
		*n*	*N*	%
Stakeholder engagement	100	112	136	82
Completeness of forms and reports	100	133	136	98
Timeliness of IESS reports	100	135	136	99
Reporting cases by source	100	128	136	94
Feedback on data transmission	100	57	128	60

**Average score**	**100**	**-**	**-**	**87**

Note: Assessment (Varkevisser) = Good.

IESS, Integrated Epidemiological Surveillance System.

#### Integrated Epidemiological Surveillance System’s sensitivity in Chad

Table 4 presents the observed scores of the sensitivity criteria. The sensitivity of the IESS in Chad is low with a score of 29% for cases reported between 2022 and 2024. Indeed, at the national level, the system is moderately sensitive, with a national rate of 36%, while for all the samples sent abroad, only 3% are true positives.

**TABLE 4:** Assessment of the sensitivity of the Integrated Epidemiological Surveillance System.

IESS sensitivity scale	Expected score (%)	Weight (%)†	Observed score (%)‡
Value of sensitivity at the national level (2022–2024)	100	79	36
Value of sensitivity abroad (2022–2024)	100	21	3

**Weighted average score**	**100**	**100**	**29**

Note: Assessment (Varkevisser) = Low.

IESS, Integrated Epidemiological Surveillance System.

† , Weight = Number of samples analysed in Chad (*N* = 1) / Total number of samples received (*N* = 2);

‡ , See Table 2-A5 in Appendix 5.

#### Positive predictive value of Integrated Epidemiological Surveillance System in Chad

Table 5 presents the observed scores of the Positive Predictive Value (PPV) criteria. The probability of correctly identifying confirmed cases (PPV) in Chad is considered low with an observed value of 29% for samples submitted between 2022 and 2024. Indeed, the national value of the PPV is not negligible (36%), while that of samples sent abroad is very low (3%).

**TABLE 5:** Assessment of the Integrated Epidemiological Surveillance System Positive Predictive Value.

IESS PPV scale	Expected score (%)	Weight (%)†	Observed score (%)‡
PPV at the national level (2022–2024)	100	79	36
Overseas PPV (2022–2024)	100	21	3

**Weighted average score**	**100**	**100**	**29**

Note: Assessment (Varkevisser) = Low.

IESS, Integrated Epidemiological Surveillance System; PPV, Positive Predictive Value.

† , Weight = Number of samples analysed in Chad (1) (See Table 2-A5 in Appendix 5 for meanings). Total number of samples received (2) (see Table 2-A5 in Appendix 5 for meaning);

‡ , See Table 2-A5 in Appendix 5.

#### Representativeness of the Integrated Epidemiological Surveillance System in Chad

Table 6 presents the observed scores of the representativeness criteria. The representativeness of the IESS in Chad is low, with an average score of 27% of the expected score. Indeed, less than half of the actors surveyed say that surveillance data are collected according to age and few mention gender (33% [*n* = 5/15]), let alone location (27% [*n* = 4/15]) and time (13% [*n* = 2/15]). These scores show that the case samples collected weakly reflect the structure of the general population.

**TABLE 6:** Assessment of the representativeness of the Integrated Epidemiological Surveillance System.

Criteria for the representativeness of the IESS	Expected score (%)	Observed score (*n* = 15)	
		*n*	%
Data are collected by age	100	5	33
Data are collected by gender	100	5	33
Data are collected by location	100	4	27
Data are collected on a time-based basis	100	2	13

**Average score**	**100**	**-**	**27**

Note: Assessment (Varkevisser) = Low.

IESS, Integrated Epidemiological Surveillance System.

#### Integrated Epidemiological Surveillance System’s responsiveness in Chad

Table 7 presents the observed scores of the reactivity criteria. The responsiveness of the IESS in Chad is considered acceptable, with an average observed score of 55% of the expected score. This assessment can be explained by its ability to collect, process and analyse data internally (57% [*n* = 76/136]) on the one hand and the system’s preparedness for possible responses in the event of an epidemic on the other. In addition, according to the agents interviewed, the IESS has an epidemiological threshold before any response (45% [*n* = 57/128]). Where applicable, they indicated that they had consultation files (95% [*n* = 129/136]), sampling sheets (85% [*n* = 109/128]) and sampling kits (63% [*n* = 86/136]). However, only 15% (*n* = 20/136) of agent’s report having a formal intervention plan and most intervention kits are not prepared (3% [*n* = 5/136]).

**TABLE 7:** Assessment of the responsiveness of the Integrated Epidemiological Surveillance System.

Responsiveness criteria of the IESS	Expected score (%)	Observed score		
		*n*	*N*	%
In-house data processing and analysis	100	76	136	57
Existence of a response plan	100	20	136	15
Existence of consultation registers	100	129	136	95
Existence of case collection cards	100	109	128	85
Existence of a response guide	100	101	136	74
Availability of prepared response kits	100	5	136	3
Existence of prepared sampling kits	100	86	136	63
On the existence of an epidemic threshold	100	57	128	45

**Average score**	**100**	**-**	**-**	**55**

Note: Assessment (Varkevisser) = Acceptable.

IESS, Integrated Epidemiological Surveillance System.

#### Quality of Integrated Epidemiological Surveillance System in Chad

Appendix 5 presents the summary of the observed scores of the indicators, as well as the overall score of the quality of the IESS. According to these results, the quality of IESS in Chad – evaluated by the CDC method – is considered low with an overall score of 44% (other pillars have been evaluated by descriptive analysis). This assessment is strongly influenced by the low sensitivity of the system (29%) and the low probability of the system correctly identifying positive cases (PPV = 29%), as well as its low representativeness (27%). Nevertheless, it should be observed that the acceptability (82%), simplicity (72%) and responsiveness (55%) of the IESS, thanks to their good or acceptable assessments, have enhanced the overall assessment of the quality of the system (Figure 1).

**FIGURE 1:**
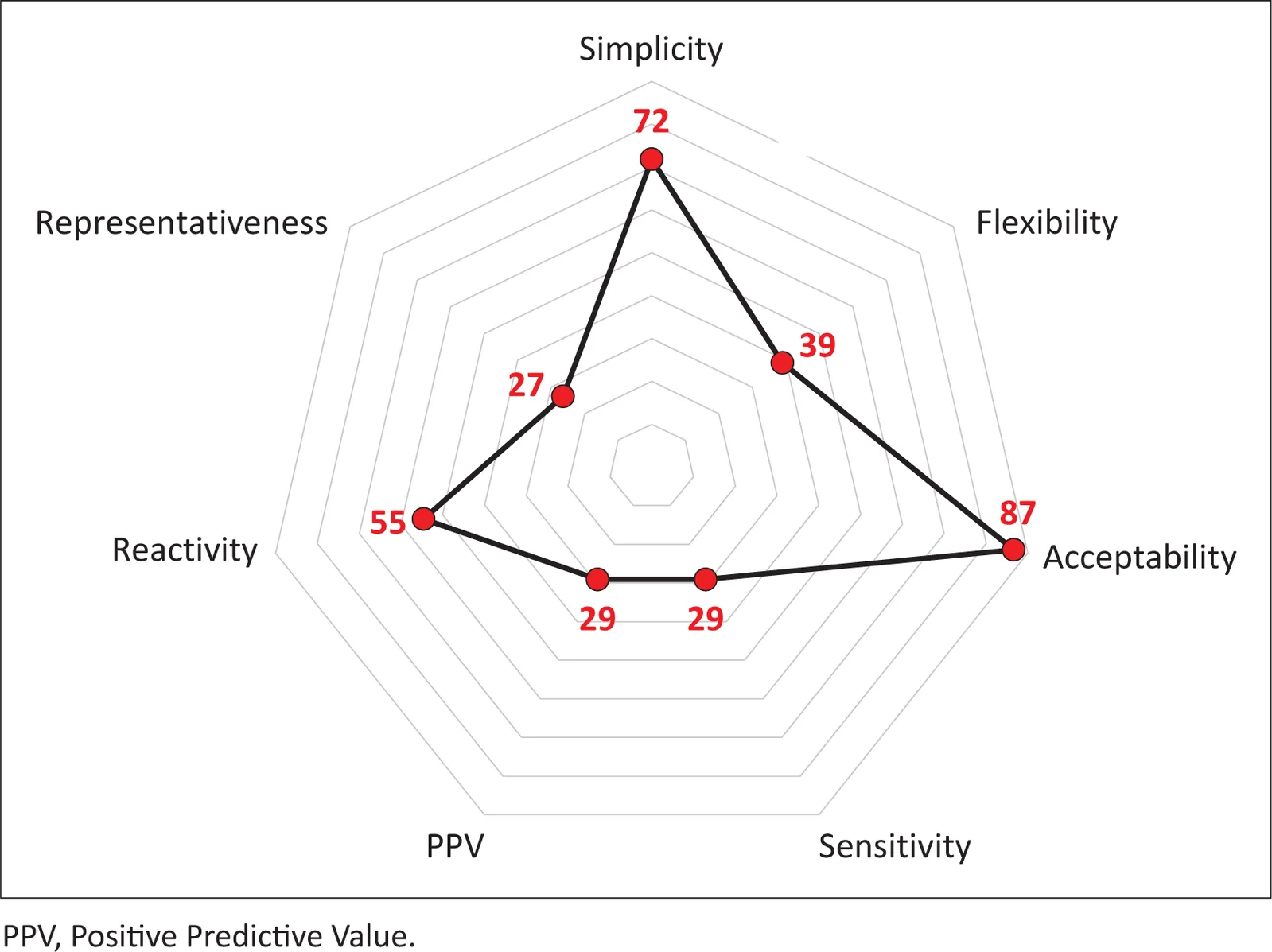
A summary of the assessment of Integrated Epidemiological Surveillance System quality criteria in Chad.

## Discussion of the results

The overall quality of the IESS in Chad (44%) appears to be low compared to that of Mali (85%).^11^ Although the simplicity, acceptability and responsiveness of this system have enabled it to maintain the minimum required, this assessment reflects the cumulative effect of the weaknesses observed in flexibility, representativeness, sensitivity and PPV.

### Acceptability and simplicity: The strengths of Integrated Epidemiological Surveillance System quality in Chad

The high acceptability of the IESS (87%) testifies to a strong commitment of the actors and a good appropriation of the system by the staff. This result, close to that of Côte d’Ivoire (93.9%)^12^ and higher than Mauritania (46%),^13^ is explained by effective management of reports (98% completeness and 99% timeliness) and capacity building efforts.^14^ Recent studies show that the completeness and timeliness of reports improves users’ perception and confidence in the system.^15,16^ Thus, this high acceptability represents an essential lever to strengthen the active participation of stakeholders and improve the overall performance of the IESS.

The simplicity of the IESS in Chad (72%) reflects an overall favourable perception of the system by users. Although this rate is lower than that observed in Côte d’Ivoire (81.3%)^12^ and Niger (86.96%),^17^ it remains higher than Mauritania’s 63%.^3^ This relatively satisfactory level is explained by the availability of clear (93%) and easily applicable case definitions, promoting good understanding among health personnel (98%).^18^ However, the complexity of mandatory laboratory confirmation is a significant constraint. As highlighted in the WHO regional report,^19^ the lack of infrastructure and equipment delays confirmations and alters the fluidity of the process. Despite these limitations, the level of simplicity observed remains encouraging in a context where technical capacity and human resources remain limited.

### The flexibility and responsiveness of the Integrated Epidemiological Surveillance System in Chad: Acceptable but to be improved

With a flexibility of 56%, the IESS in Chad has a moderate capacity to adapt, lower than the results reported in Côte d’Ivoire (96.8%),^12^ but slightly higher than those of Mauritania (46%).^13^ This situation is because of several factors: the lack of qualified personnel, the weakness of the information technology (IT) and laboratory infrastructure, logistical and administrative difficulties, as well as limited and irregular funding.^19,20,21^ These constraints would slow down the evolution of the system in the face of new needs and reduce its operational flexibility. The IESS nevertheless has the potential to adapt, but it remains hampered by structural and organisational problems that require sustained support.

The responsiveness of the IESS (55.0%) remains below the results recorded in Togo (93.01%) or Burkina Faso (93.2%).^22^ This underperformance stems from the lack of logistical, communication and operational resources, as highlighted in the WHO Cooperation Strategy for Chad (2024–2027).^23^ These weaknesses lead to delays in reporting, investigating and confirming cases. While this value can be considered average in the national context, it reveals the need for system strengthening to respond more effectively to health emergencies.

### Sensitivity, PPV and representativeness: the weak points of the Integrated Epidemiological Surveillance System in Chad

The sensitivity of the IESS in Chad (29%) is particularly low compared to that of Niger (100%).^2^ This observation is in line with the observations of the Red Cross and the WHO, which highlight the weak capacity of the Chadian system to detect and confirm epidemic alerts.^24,25^ The main causes are lack of training, insufficient logistics and transport, as well as weak coordination.^26^ These limitations restrict early detection and escalation, compromising overall sensitivity. A targeted reinforcement of training, resources and logistics is therefore essential to improve this key component of the IESS.

The observed PPV (29%) reflects a low capacity of the IESS to confirm actual cases among suspects. This rate is much lower than those of Togo (64%)^12^ and Ghana (57%),^27^ but remains comparable to those of Burkina Faso (22%)^28^ and Nigeria (22%).^29^ This weakness is mainly because of a lack of training leading to the weak application of clinical criteria, and overly broad case definitions,^30,31^ and the lack of laboratory equipment to confirm cases.^32^ Despite this limited performance, the result highlights priority areas for improvement: strengthening diagnostic skills, revising case definitions and improving the availability of confirmation tools.

With an estimated 27.0% representativeness, the Chadian IESS struggles to effectively cover the entire territory. This rate, lower than that observed in Togo (37.8%),^12^ is because of difficulties in accessing rural areas, weak road conditions and inadequate logistical and communication facilities.^33,34^ These constraints limit the frequency and quality of reports, reducing the coverage and reliability of the data collected. As a result, the limited representativeness compromises the ability of the IESS to provide a complete picture of the national epidemiological situation.

### Functioning, importance, usefulness, cost and quality of the Integrated Epidemiological Surveillance System in Chad

#### Functioning and quality of the Integrated Epidemiological Surveillance System in Chad

The low quality of the IESS in Chad is evident from the description of its operation. Indeed, the standardised case definition is not available for all priority diseases, let alone in all health facilities. In addition, the analysis and interpretation of surveillance data is often only performed at the central level, which could be a bottleneck at the peripheral level for the information system and the responsiveness of the IES at the peripheral level. In addition, the high dependence on the WHO and/or the Centre Pasteur de Yaoundé in Cameroon and/or the Institut Pasteur in Dakar, Senegal, exposes the IESS in Chad to a risk of reactivity for epidemics that spread rapidly and with high lethality but also in the face of epidemics requiring travel restrictions, as was the case during the COVID-19. In addition, feedback remains very low within the IESS in Chad, which could explain the limits of responsiveness observed. Finally, since its establishment in 2001, only two evaluations have been carried out (2011 and 2024). The functioning of the IESS in Chad does not provide for a regular evaluation (internal or external) to identify any dysfunctions and adjustments. Several studies highlight similar issues in epidemiological surveillance systems in Africa, including excessive centralisation, lack of feedback, dependence on external partners, and a lack of regular evaluations – as confirmed by the reports of the WHO Joint External Assessment of the Republic of Chad’s Key RSI Capabilities (JEE), reviews published in BioMed Central (BMC) Public Health and Public Library of Science (PLoS) One, and benchmarking in Rwanda and Senegal, which illustrate more autonomous and responsive approaches.^35,36,37,38^

#### Usefulness, importance and quality of the Integrated Epidemiological Surveillance System in Chad

The answers to the eight questions of usefulness of an Epidemiological Surveillance System (ESS) of Dabis et al.,^10^ show that the IESS in Chad is indeed useful. However, this usefulness is confronted with the opacity in financial management and the absence of an impact assessment report on measures to prevent or control health problems. In addition, between 2021 and 2024, the contrasting trends in communicable and non-communicable diseases – marked by outbreaks of measles and diphtheria, a persistent burden of malaria, environmental emergencies, worrying maternal and child indicators, and the emergence of new pathologies – underscore the urgent need to strengthen immunisation, epidemiological surveillance, basic healthcare, and the health system’s response capacity to a global constantly changing. Several studies, such as that of Noubaramadje on the health system in Chad (8th European Development Fund (EDF), 2006–2008), the National Strategy for the Promotion of Health and Well-being (2016–2020), and recent projects to improve the quality of maternal and newborn care (2023), confirm the theoretical usefulness of the IESS while highlighting persistent limitations in transparency, impact assessment and responsiveness to epidemiological developments, thus joining the findings of this study.^39,40,41^

#### Operating cost and quality of the Integrated Epidemiological Surveillance System in Chad

As confirmed by several studies,^42,43^ the quality of the IESS is hampered by deficiencies in information systems, the lack of qualified human resources, the lack of adequate equipment and logistics, and insufficient sustainable funding. These findings are consistent with the CDC’s evaluation model,^44^ which highlights the importance of these dimensions in overall performance. The results of this work show that these three dimensions characterise the IESS in Chad. Indeed, all regions have expressed an urgent need for additional staff. Although Ndjamena is an exception in this regard, the staff there have expressed a need for training or capacity building on Integrated Epidemiology Surveillance System (IESS) techniques and tools. In addition, although we have noted the availability of collection tools, sample collection kits and cold chains, the need for means of communication and travel remains very great and urgent. Also, despite the opacity that we have observed in the financial management of the IESS in Chad, we have been able to observe the glaring and urgent lack of funding and a great opacity in the management of the fine part made available. Indeed, less than 1% of the MPHP’s budget is allocated to the IESS and almost all the agents surveyed reported arrears of bonuses, sometimes dating back more than a year at the time of the survey. In addition, the total cost of the IESS’s activities remains incalculable because of the lack of communication, let alone the sharing of information on financial aspects despite requests at both the central and provincial levels.

### Limitations of the study

Quantitative cross-sectional research leads to not only a selection bias because of refusals or illness or even death, but also to a bias of human feelings that are not categorisable. This is the case of illness before and during the investigation of some focal points who could not be questioned either. Also, six clinicians from private structures (clinics, doctors’ surgeries, care practices) were not surveyed because they had never benefited from visits from the surveillance (sites selected for surveillance but not visited). In addition, the respondents may not have been completely sincere in their answers, fearing that they will give a bad image of their structure and themselves. In the field, we had not always been able to access all the information necessary for a comprehensive assessment of each attribute of the CDC model, particularly those concerning the financing of Epidemiological Surveillance (ES) activities.

### Validity of the study and management of potential bias

Cross-sectional studies are prone to selection and information bias. In this case, the selection of DPSs and DSs by reasoned choice could constitute sources of inherent bias. Indeed, the absence of a sampling frame – notably a comprehensive and up-to-date register of all IESS staff at national level – made it impossible to carry out a rigorous probabilistic sample; nevertheless, these risks were mitigated on at least three levels:

- A degree of geographical randomisation was nevertheless maintained by selecting 8 out of 23 Provincial Delegations at random.
- Strict inclusion criteria (residence of more than 12 months, active participation in the IESS, informed consent) limited information bias.
- Both urban and rural areas were considered when selecting respondents to ensure a diversity of information across different settings.

These three measures are designed to reduce the selection and information biases that may be associated with data sources and survey respondents. This approach is consistent with similar studies published in Mali, Niger and Senegal.^15,17,38^

### Main recommendations and lessons for countries in the sub-Saharan region

In view of the results obtained and discussed, we recommend that the MPHP and national and international partners, both at the central and peripheral levels:

- Equip provincial hospitals with laboratory kits, in quantity and quality, necessary for the confirmation of cases at the provincial level.
- Strengthen the capacity of the ES central laboratory to confirm AFP cases at the national level.
- Conduct awareness campaigns for the community about compliance with the preventive measures taken by the health authorities in the event of an epidemic.
- Strengthen the capacity of Head of the Epidemiological Surveillance Unit (HESU), Focal Point (FP) and clinicians on case definition (diagnostic) and procedures for the collection and safe transport of samples, both nationally and internationally.
- Consider the characteristics of representativeness (time, place, sex, age) in the Intrusion Detection System (IDS) database.
- Pre-provision comprehensive disease response kits under surveillance in all health facilities.
- Produce monthly feedback of laboratory data and results, in the form of bulletins at all levels.
- Hold regular biannual monitoring and evaluation meetings (review meeting) with health delegates, district medical officers, branch heads and FPs.
- Mobilise and allocate financial resources, communications and travel to provincial and district surveillance services.
- Order reagents and consumables for IES’s two sample analysis laboratories.
- Pay the air of fuel costs and allowances for 2024 (or before) of all actors involved in surveillance activities.
- Improve the physical and electronic archiving (servers or secure cloud) of records and databases.
- Regularly update the list of priority sites considering private actors and strengthening community-based surveillance by involving key informants.
- Promote public-private partnership in IES.
- Develop and implement action plans integrating surveillance at the provincial and health districts (HD) levels.
- Strengthen supplementary immunisation activities (SIA) campaigns and routine EPI by focusing on the Active Cases Detection (ACD) approach and the fixed, advanced and mobile strategy, or even door-to-door.

Table 1-A6 in Appendix 6 presents the details of the recommendations by level of responsibility, the problems identified and the target targeted.

There are four main lessons, that sub-Saharan countries could draw from those results:

- Applying the five pillars of the CDC model enables a broader systemic assessment of IESS in resource-constrained countries, which is essential given that partial approaches are insufficient to account for all structural failures.
- Low sensitivity and PPV, combined with very low representativeness, constitute a structural factor in underperformance, regardless of the level of acceptability, because the commitment of health workers cannot compensate for deficiencies in the diagnosis or infrastructure.
- Dependence on external laboratories constitutes a major operational risk in crisis situations, as COVID-19 has demonstrated. Strengthening national biological platforms and subregional pooling of analytical capacities must be among the strategic priorities.
- The chronic underfunding of the IESS (less than 1% of the Ministry of Health’s budget) and the delayed payment of staff bonuses (a finding also observed in comparable studies in Mali, Mauritania, and Côte d’Ivoire) are determinants that must be addressed to improve the IESS’s poor performance.

## Conclusion

The objective of this study was to evaluate the IESS in Chad, using the five pillars of the CDC Atlanta method, namely the functioning, usefulness, importance, cost and quality of the surveillance system. The analysis of data collected from 16 September 2024 to 15 January 2025 on a sample of IESS agents in representative districts, reveals a low quality (44%) of the IESS in Chad. Indeed, while simplicity (72%) and acceptability (87%) attest to a real commitment of the actors and a relative ease of use of the system, the sensitivity (29%), the PPV (29%), the representativeness (27%) paradoxically reveals shortcomings in the detection, notification and management of health events. In addition, the strengthening of IESS in Chad – and therefore its importance – is more urgent than ever because of the resurgence (cases and/or fatality) of epidemics that were once under control such as measles, the persistence of epidemics such as malaria, and the appearance of epidemics such as mpox and dengue in 2024. The results of this work also show that three dimensions of the cost of the IESS in Chad justify its low quality: the glaring and urgent need for qualified human resources, adequate equipment and logistics, as well as sufficient, sustainable and transparently managed funding. These results led to several recommendations depending on the level of responsibility. Indeed, in order to guarantee early detection and a rapid, effective and sustainable response to diseases under surveillance, the strengthening of the IESS in Chad requires the improvement of diagnostic capacities at both the provincial and central levels; the mobilisation of financial, logistical and human resources; the implementation of integrated action plans; and the motivation of the actors through the regular payment of their allowances and fuel costs. In addition, particular attention should be paid to data quality, feedback, multisectoral coordination and community involvement. Also, the strengthening of preventive actions in the fight against vaccine-preventable diseases, especially in routine EPI, and compliance with hygiene and health measures.

### Contribution to existing knowledge

Six main contributions of this study are:

- We used one of the most rigorous, comprehensive, internationally standard evaluation methods (the Atlanta CDC model) to ensure the objectivity, reliability and validity of the results.
- Most similar work, using the CDC method, has been limited to the evaluation of the quality of the surveillance system. In this study, we have taken care to analyse the five pillars of the CDC model, including the functioning, usefulness, importance, cost of the IESS and the quality of the IESS in Chad.
- We rigorously quantified seven criteria out of eight of the quality of the IESS in Chad compared to four on average in the literature.
- We determined the current overall performance (2024) of the IESS in Chad.
- We identified the strengths and weaknesses of the quality of IESS in Chad.
- We made several recommendations based on the issues identified, the level of responsibility and the intended target.

## Figures and Tables

**FIGURE 1-A3:**
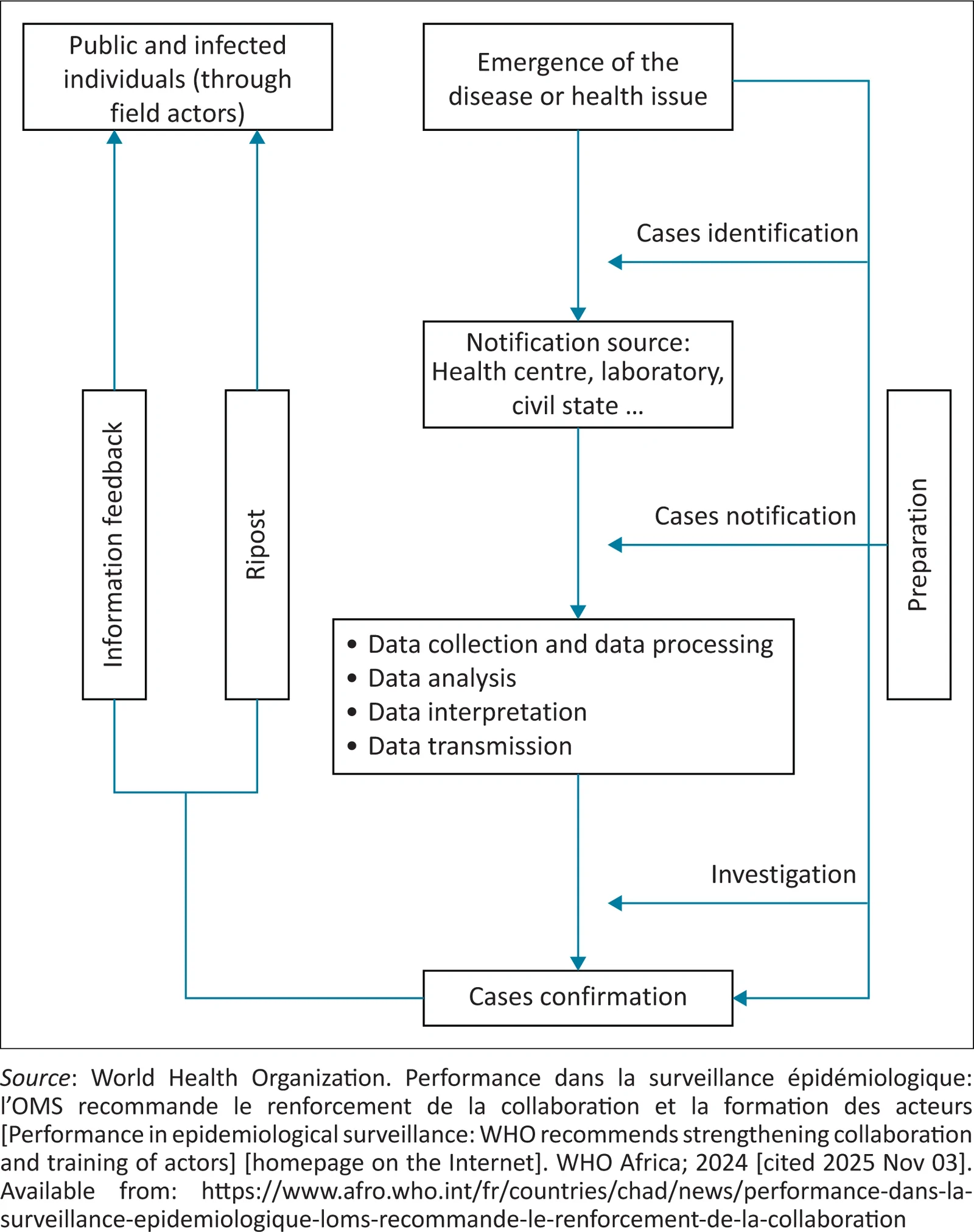
Block diagram of the Integrated Epidemiological Surveillance System in Chad.

**FIGURE 1-A4:**
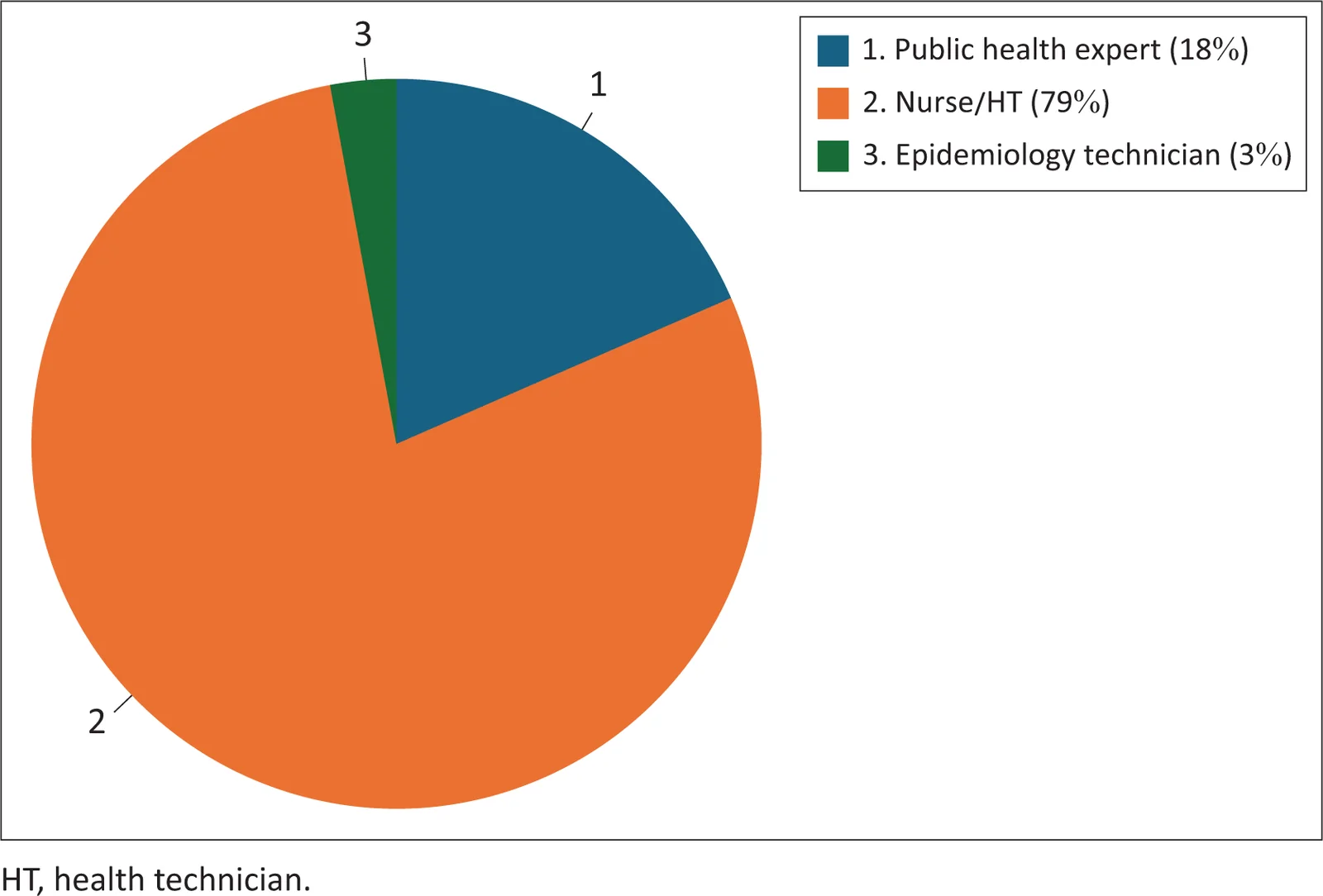
Distribution of respondents by qualification requested (*n* = 105).

**FIGURE 2-A4:**
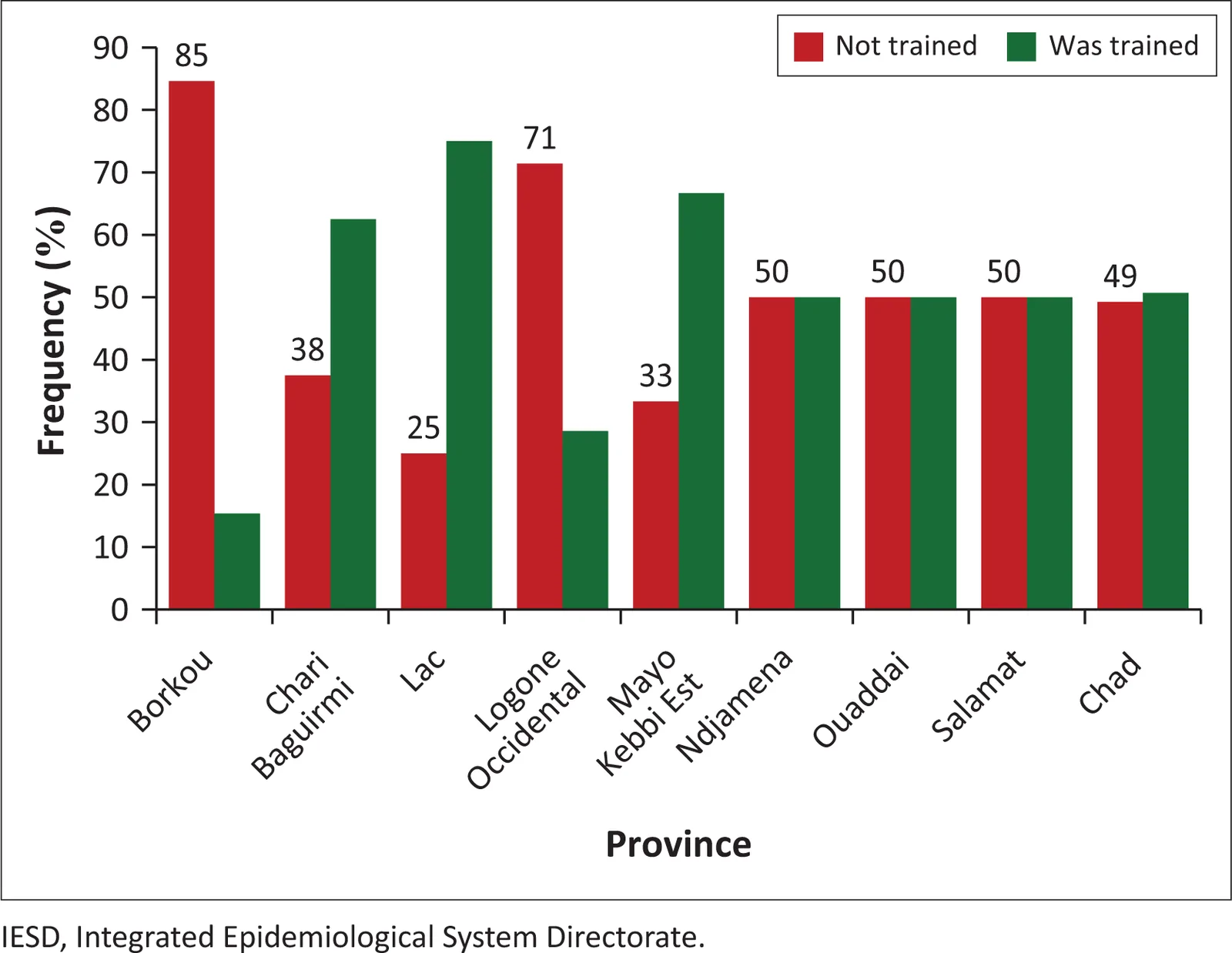
Distribution of IESD-trained respondents by province.

**FIGURE 3-A4:**
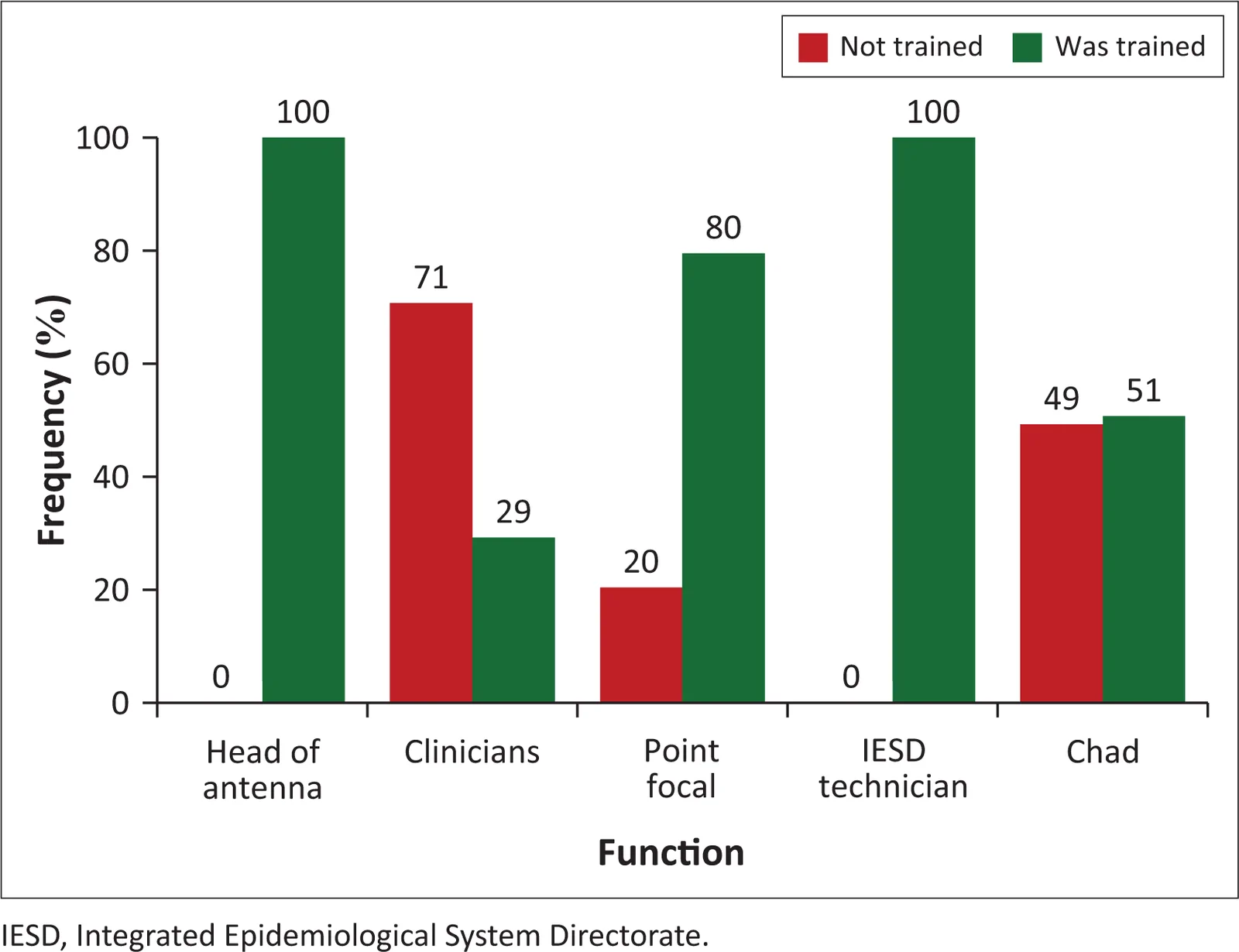
Distribution of IESD-trained respondents by function.

**FIGURE 4-A4:**
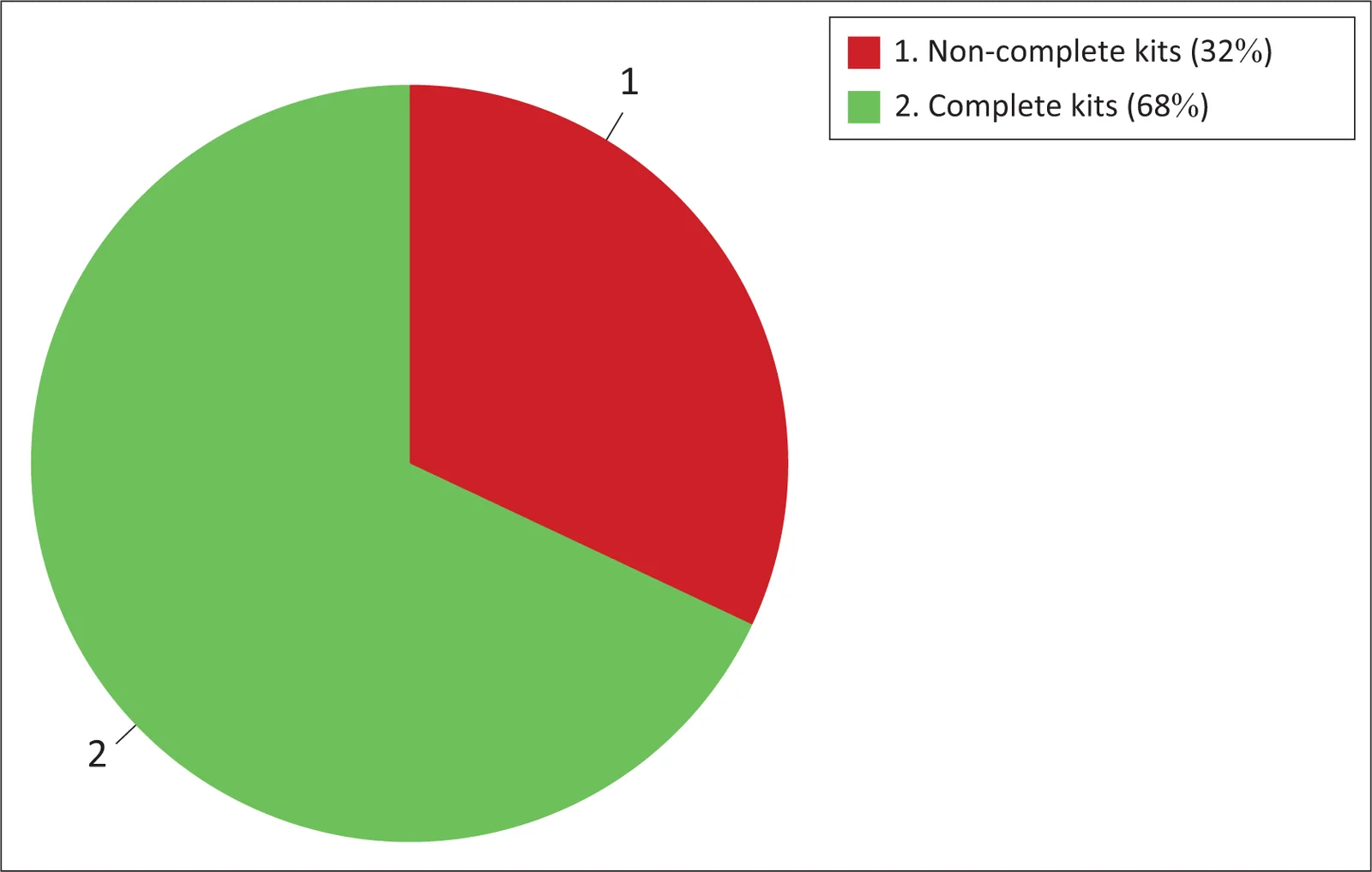
Distribution of respondents by completeness of available surveillance kits (*n* = 119).

**FIGURE 5-A4:**
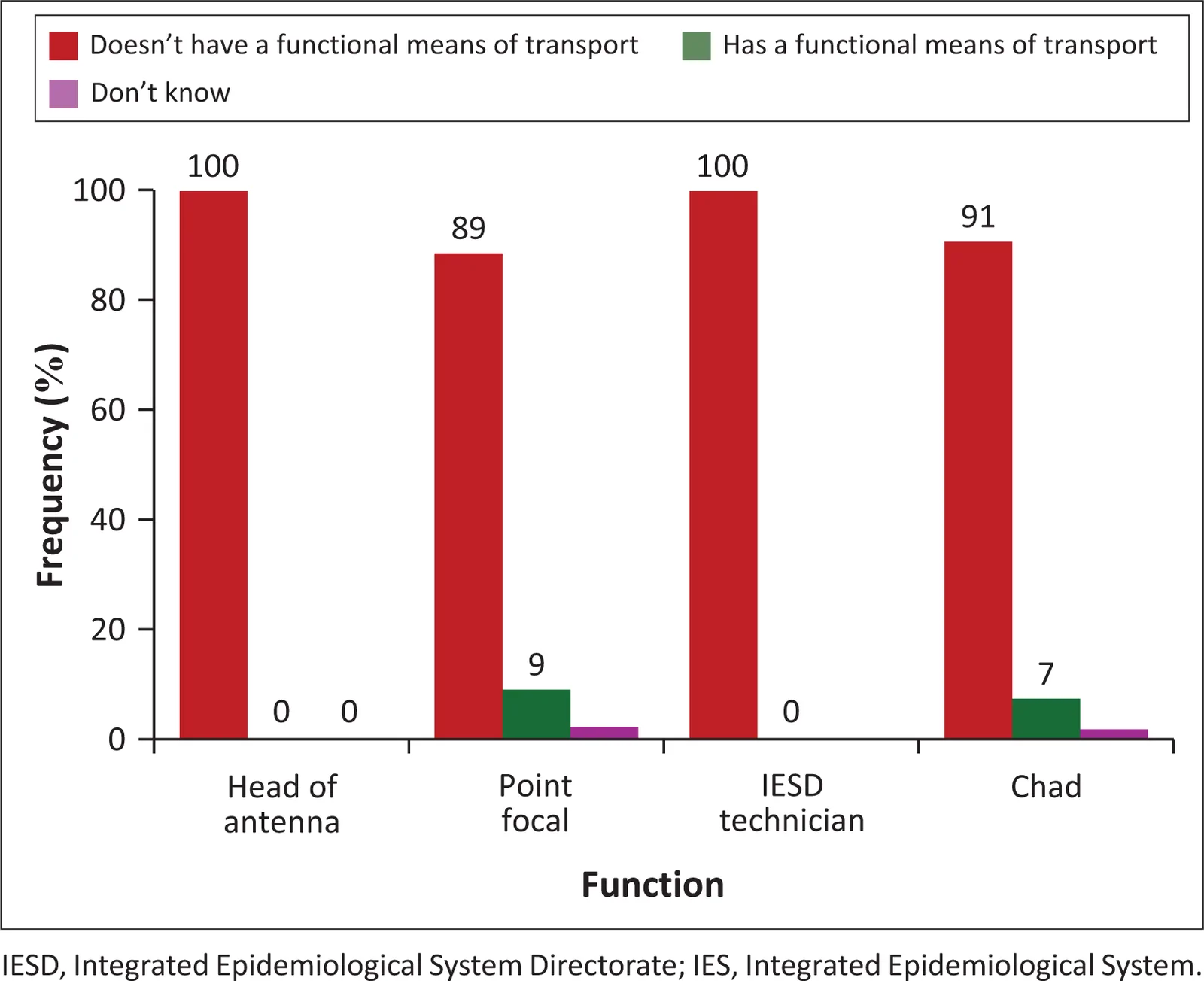
Distribution of respondents by availability of functional IES travel by function.

**FIGURE 6-A4:**
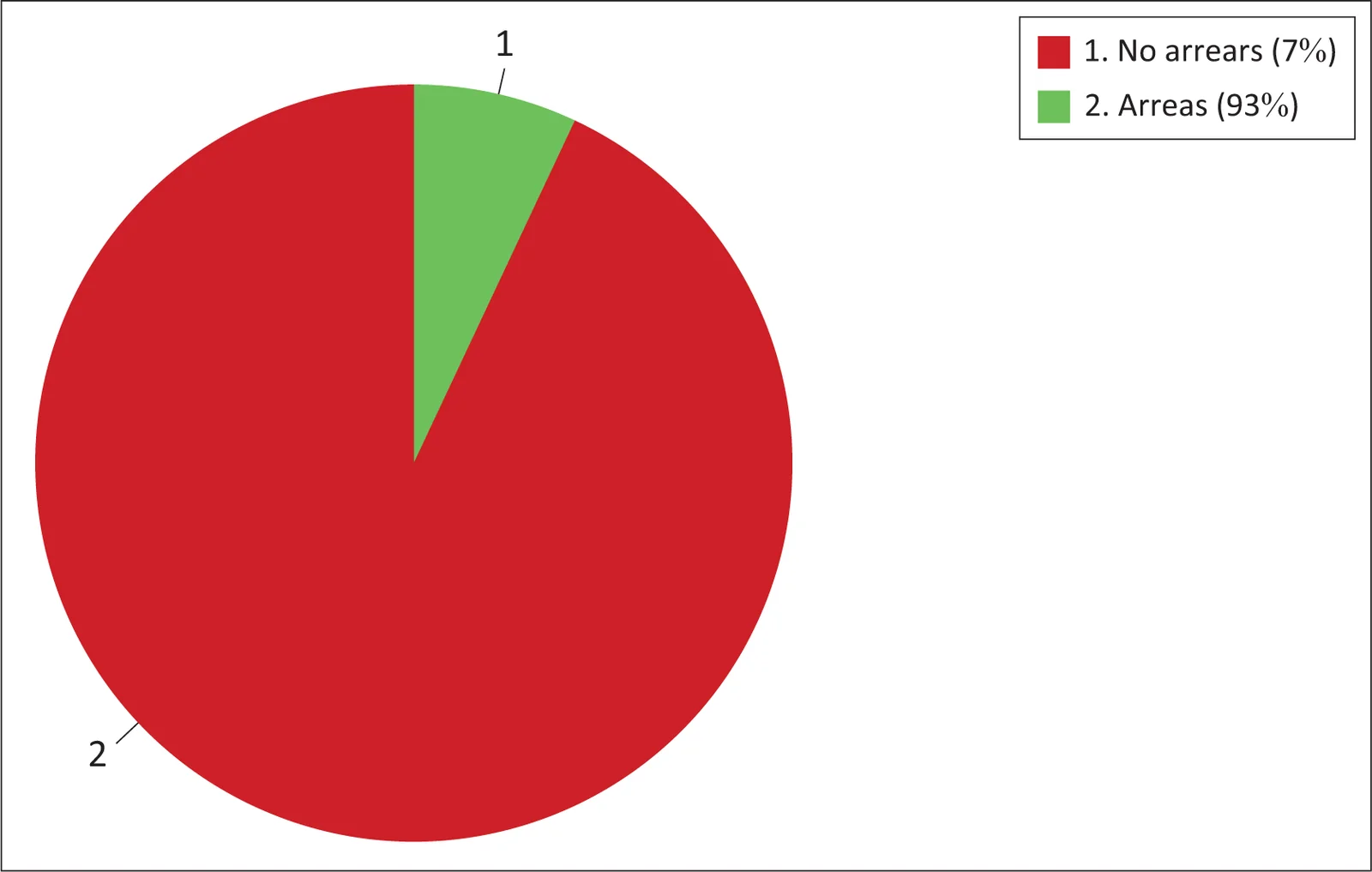
Incentive arrears and fuel costs for Integrated Epidemiology Surveillance (IES).

## Appendix 1

**TABLE 1-A1:** Indicators and criteria for assessing the quality of the Integrated Epidemiological Surveillance System in Chad.

Indicators and criteria		Description	Source	Expected score (%)
**Simplicity of the ESS**				**100**
1	Time to confirmation of cases	Timeliness rate of IESS reports	Field survey	100
2	Existence of the case definition	Proportion of officers who report the existence of the case definition	Field survey	100
3	Knowledge of case definition	Proportion of officers who are aware of the case definition	Field survey	100
4	Mandatory laboratory confirmation of cases	Feedback rate of results sent to the laboratory	Field survey	100
5	Dependence on related services	Proportion of agents who process and analyse data internally	Field survey	100
**Flexibility of the ESS**				**100**
1	Flexibility Induced by Simplicity	1/8 or 13% if simplicity is at least acceptable and 0% otherwise	Field survey (Simplicity indicator score)	13
2	Has not encountered any difficulties in adapting during the last epidemic (case of COVID-19)	1/8 or 13% if yes 0 otherwise	Evaluation reports of the ESS of Chad during and after the COVID-19 (MPHP, WHO Chad, UNDP)	13
3	Total coverage of access to care throughout the country.	1/8 or 13% if there was no inequality in access to care during COVID-19 and 0 otherwise	Field survey	13
4	Unlimited emergency response capacity	1/8 or 13% if the notification, detection and management during the COVID-19 were prompt and complete 0 otherwise	Evaluation reports of the ESS of Chad during and after the COVID-19 (MPHP, WHO Chad, UNDP)	13
5	Availability of medical resources	1/8 or 13% if qualified personnel, as well as adequate vaccines and materials were available in quantity, especially for urgent cases and 0 otherwise	Evaluation reports of the ESS of Chad during and after the COVID-19 (MPHP, WHO Chad, UNDP)	13
6	Coordination and weak governance	1/8 or 13% if there has been a great deal of collaboration between health institutions and international partners and 0 otherwise	Evaluation reports of the ESS of Chad during and after the COVID-19 (MPHP, WHO Chad, UNDP)	13
7	Responsiveness of the information system	1/8 or 13% if the epidemiological data collected during COVID-19 were complete and collected in real time and 0 otherwise	Evaluation reports of the ESS of Chad during and after the COVID-19 (MPHP, WHO Chad, UNDP)	13
8	Community Membership	1/8 or 13% if the exposed communities were in line with the IESS by putting into practice the social distancing and hygiene measures issued during the COVID-19 and 0 otherwise	Evaluation reports of the ESS of Chad during and after the COVID-19 (MPHP, WHO Chad, UNDP)	13
**Acceptability of the ESS**				**100**
1	Stakeholder participation	Community participation rate in IES activities	Field survey	100
2	Completeness of IES Forms and Reports	Proportion of complete forms and reports	Field survey	100
3	Timeliness of IES Reports	Timeliness rate of IES reports	Field survey	100
4	Reporting of cases by source	Case reporting rates by source	Field survey	100
5	Feedback on data transmission	Feedback rate on data transmission	Field survey	100
**Sensitivity of the ESS**				**100**
1	National sensitivity	Value of sensitivity on samples analysed centrally between 2022 and 2024	Field survey	100
2	Sensitivity abroad	Value of sensitivity on samples analysed abroad between 2022 and 2024	Field survey	100
**Positive predictive value (PPV) of ESS**				**100**
1	National PPV	Value of PPV at the central level between 2022 and 2024	Field survey	100
2	PPV Abroad	Value of the PPV abroad between 2022 and 2024	Field survey	100
**ESS responsiveness**				**100**
1	In-house data processing and analysis	Proportion of agents who report that data processing and analysis is performed in-house	Field survey	100
2	Existence of a response plan	Proportion of officers who report that there is a response plan	Field survey	100
3	Existence of consultation registers	Proportion of staff who report that consultation records exist	Field survey	100
4	Existence of case collection sheets	Proportion of officers who report that case collection sheets exist	Field survey	100
5	Existence of a response guide	Proportion of agents who report that there is a response guide	Field survey	100
6	Existence of prepared response kits	Proportion of agents who report that response kits are available	Field survey	100
7	Existence of prepared sampling kits	Proportion of agents who report that there are collection kits	Field survey	100
8	Existence of an epidemic threshold	Proportion of agents who report that there is an epidemiological threshold	Field survey	100
**Representativeness of the ESS**				**100**
1	Data are collected by age	Proportion of data collected by age	Field survey	100
2	Data are collected by gender	Proportion of data collected by sex	Field survey	100
3	Data are collected by location	Proportion of data collected by location	Field survey	100
4	Data are collected on a time-based basis	Proportion of data collected by time	Field survey	100
**Quality of the IESS**				**100**

IESS, Integrated Epidemiological Surveillance System; IESD, Integrated Epidemiological System Directorate; ESS, Epidemiological Surveillance System; COVID, coronavirus disease 2019; PPV, Positive Predictive Value; MPHP, Ministry of Public Health and Prevention; WHO, World Health Organisation; UNDP, United Nation of Development Program; IES, Integrated Epidemiological System.

## Appendix 2

**TABLE 1-A2:** Socio-demographic and occupational characteristics.

Characteristic	*n* (*n* = 136)	Percentage (%)
**Respondent profile**		
Clinician	82	60
Focal point	44	32
Anchor	8	6
IESD technician	2	1
**Sex**		
Man	117	86
Wife	19	14
**Level of education**		
Primary	1	1
Secondary	112	82
Upper	23	17
**Occupational status**		
Official	106	78
MPHP contractor	24	18
Community contract	6	4
**Service status**		
Public	119	88
Private	11	8
Parapublic	6	4
**Seniority**		
Less than a year	18	13
1 year to 4 years	65	48
5 years or more	53	39

IESD, Integrated Epidemiological System Direction; MPHP, Ministry of Public Health and Prevention.

## Appendix 3: Operation, usefulness and importance of the Integrated Epidemiological Surveillance System in Chad

**TABLE 1-A3:** Answers to the eight questions of usefulness of the Epidemiological Surveillance System in Chad.

Question	Answer
1. Does the surveillance system detect trends in the occurrence of the health problem?	Yes
2. Does it make it possible to detect epidemic episodes?	Yes
3. Does it provide reliable numerical estimates of the importance of the health problem in terms of morbidity and mortality?	Yes
4. Does it provide useful data for the initiation of applied epidemiological research programmes? Does it make it possible to formulate hypotheses in the field of prevention and control?	Yes
5. Does it identify risk factors for the occurrence of the health problem?	Yes
6. Does it contribute to the planning and allocation of resources committed to a prevention or control programme?	Don’t know
7. Can it be used to measure the impact of measures to prevent or control the health problem?	No
8. Can it be used to improve the practice of health care providers serving as informants in this surveillance system?	Yes

*Source*: Dabis F, Drucker J, Moren A. Master Sciences, Technologies, Santé, Mention Santé Publique, 2009–2010: Épidémiologie d’intervention [Master of science, technology, health, public health, 2009–2010: Intervention epidemiology]. Bordeaux: Université Victor Segalen Bordeaux 2, ISPED; 2010.

**TABLE 2-A3:** Evolution of priority diseases and events under integrated epidemiological surveillance in Chad from 2021 to 2024.

Number	Diseases	Year							
		2021		2022		2023		2024	
		Case	Death	Case	Death	Case	Death	Case	Death
01	Meningitis	308	40	236	29	197	10	291	23
02	Measles	2577	18	3134	12	10 732	8	7772	27
03	Cholera	0	0	0	0	0	0	0	0
04	AFP	919	0	1191	0	1294	0	1084	0
05	NNT	238	73	269	89	315	61	275	71
06	Yellow Fever	1103	58	1403	16	1110	10	1585	9
07	Malaria	1 018 244	1713	1 229185	1489	1 342 176	1163	1 439 999	1877
08	Malnutrition	598 510	280	697 492	256	784 430	341	942 165	666
09	Maternal death	-	313	-	356	-	260	-	385
10	Ebola fever	0	0	0	0	0	0	0	0
11	Hepatitis E	376	2	132	0	1	0	2099	7
12	Scorpion sting	8503	18	8699	52	9937	85	10 661	70
13	Guinea worm	7	0	6	0	0	0	0	0
14	Human Influenza	1	0	363	0	0	0	0	0
15	Chikungunya	0	0	0	0	0	0	0	0
16	Bite/snake	3350	22	3219	32	2847	26	3088	25
17	COVID-19	-	-	179	3	167	0	0	0
18	Dengue fever	-	-	-	-	-	-	17	0
19	Diphtheria	-	-	-	-	-	-	2595	70
20	Mpox	-	-	-	-	-	-	26	1
21	Dog bite	-	-	-	-	31	0	16	0

Note: Table data from IDS (Intrusion Detection System) databases of Chad’s IESS (Integrated Epidemiological Surveillance System).

AFP, acute flaccid paralysis; NNT, Neonatal tetanus; COVID, coronavirus disease 2019; IESD, Integrated Epidemiological System Directorate.

## Appendix 4: Cost of Running Integrated Epidemiological Surveillance System in Chad

**TABLE 1-A4:** Human resource availability of Chad’s Integrated Epidemiological Surveillance System in 2024.

Human resources	Borkou (*n* = 13)		Chari Baguirmi (*n* = 16)		Lake (*n* = 16)		Logone Occidental (*n* = 14)		Mayo Kebbi East (*n* = 15)		Ndjamena (*n* = 30)		Ouaddaï (*n* = 16)		Salamat (*n* = 16)		Chad (*n* = 136)	
	*n*	%	*n*	%	*n*	%	*n*	%	*n*	%	*n*	%	*n*	%	*n*	%	*n*	%
**Need for additional staff**																		
Yes	92	12	75	12	100	16	93	13	80	12	47	14	81	13	81	13	77	105
No	8	1	25	4	0	-	7	1	20	3	97	29	19	3	19	3	23	31
**Personnel trained on the IESS**																		
Yes	15	2	63	10	75	12	29	4	67	10	50	15	50	8	50	8	51	69
No	85	11	38	6	25	4	71	10	33	5	50	15	50	8	50	8	49	67

IESS, Integrated Epidemiological Surveillance System.

**TABLE 2-A4:** Availability of material and logistical resources.

Material and logistical resources	Borkou (*n* = 13)		Chari Baguirmi (*n* = 16)		Lake (*n* = 16)		Logone Occidental (*n* = 14)		Mayo Kebbi East (*n* = 15)		Ndjamena (*n* = 30)		Ouaddaï (*n* = 16)		Salamat (*n* = 16)		Chad (*n* = 136)	
	%	*n*	%	*n*	%	*n*	%	*n*	%	*n*	%	*n*	%	*n*	%	*n*	%	*n*
**Availability of collection tools (weekly bulletins, case investigation sheets)**																		
Yes	92	12	94	15	88	14	93	13	93	14	70	21	88	14	100	16	**88**	**120**
No	8	1	6	1	12	2	7	1	7	1	30	9	12	2	0	**12**	**16**	**-**
**Availability of monitoring sample collection kits**																		
Yes	92	12	94	15	88	14	93	13	93	14	70	21	88	14	100	16	**88**	**120**
No	8	1	6	1	12	2	7	1	7	1	30	9	12	2	0	-	**12**	**16**
**Availability of a means of travel for monitoring**																		
Yes	0	-	0	-	14	2	20	1	0	-	9	3	12	2	0	-	**7**	**10**
No	100	13	100	16	86	14	80	11	100	16	91	27	88	14	100	16	**93**	**126**
**Availability of communication tools for data transmission**																		
Yes	100	13	100	16	94	15	86	12	93	14	93	28	94	15	82	13	**93**	**126**
No	0	-	0	-	6	1	14	2	7	1	7	2	6	1	18	3	**7**	**10**
**Availability of a cold chain**																		
Yes	100	13	87	14	75	12	86	12	100	13	93	28	100	16	100	16	**94**	**128**
No	0	-	13	2	25	4	14	2	0	-	7	2	0	-	0	-	**6**	**8**

**TABLE 3-A4:** Budget allocated to the Integrated Epidemiological System by the MPHP.

Year	Total budget of the MPHP (FCA)	Budget allocated to the IESS (FCA)	Proportion (%)
2022	74 359 010 027	6 000 000	0.81
2023	79 536 476 625	5 500 000	0.69
2024	124 268 516 602	7 500 000	0.60

Note: CFA represent Communauté Financière Africaine [African Financial Community francs].

IESS, Integrated Epidemiological Surveillance System; MPHP, Ministry of Public Health and Prevention; CFA, Communauté Financière Africaine [African Financial Community].

**TABLE 4-A4:** Incentive arrears and fuel costs by number of months.

Provinces	No arrears	1–6 months	7–12 months	13–24 months	Total
Borkou	1	0	3	0	4
Chari Baguirmi	0	1	1	5	7
Lake	0	0	3	4	7
Logone Occidental	0	0	3	2	5
Mayo Kebbi East	0	0	4	2	6
N’Djamena	2	0	1	8	11
Ouaddaï	1	0	3	3	7
Salamat	0	0	3	4	7

**Total**	**4**	**1**	**21**	**28**	**58**

Note: Provinces total: No arrears = 7%; 1–6 months = 2%; 7–12 months = 39%; 13–24 months = 52%; and Total = 100%.

## Appendix 5: Evaluation of the Quality of the Integrated Epidemiological Surveillance System

**TABLE 1-A5:** Evaluation of the Integrated Epidemiological Surveillance System quality criteria in Chad.

Indicators	Expected score (%)	Average score observed (%)	Assessment (Varkevisser)
Simplicity	100	72	Acceptable
Flexibility	100	39	Low
Acceptability	100	87	Good
Sensitivity	100	29	Low
PPV	100	29	Low
Responsiveness	100	55	Acceptable
Representativeness	100	27	Low

**Quality of the IESS**	**100**	**44** †	**Low**

IESS, Integrated Epidemiological Surveillance System; PPV, Positive Predictive Value.

† , Overall score = weighted average of the average scores of the indicators (cf. ‘Data processing and analysis’ section).

**TABLE 2-A5:** Estimated sensitivity and Positive Predictive Value between 2022 and 2024.

Year	Samples received by the central level				
	Total	Compliant samples	Non-compliant samples	% Conforming samples	% Non-compliant samples
2022	8101	2571	5530	31.74	68.26
2023	5847	3989	1858	68.22	31.78
2024	4752	3287	1465	69.17	30.83

**Total**	**18 700 (2)**	**9847**	**8853**	**52.66**	**47.34**

Note: See Table 5 for meaning of (2).

**TABLE 3-A5:** Estimated sensitivity and Positive Predictive Value between 2022 and 2024.

Year	Total	Positive results	Negative results	% Positive results	% Negative results
**Samples analysed at the Central level**					
2022	6846	2800	4046	**40.90**	59.10
2023	4349	1305	3044	**30.01**	69.99
2024	3556	1155	2401	**32.48**	67.52
**Total**	**14 751** (1)	**5260**	**9491**	**35.66**	**64.34**
**Samples analysed abroad**					
2022	1255	40	1215	**3.19**	96.81
2023	1498	53	1445	**3.54**	96.46
2024	1196	38	1158	**3.18**	96.82
**Total**	**3949**	**131**	**3818**	**3.32**	**96.68**

Note: Boldface values represent PPV estimated over time (2022-2024) and overall on the three years, in Chad and abroad. Those values are boldfaced because they were used to estimate the global PPV by the CDC method. See Table 5 for meaning of (1).

## Appendix 6: Assessment of the Quality of the Integrated Epidemiological Surveillance System

**TABLE 1-A6:** Assessment of Integrated Epidemiological Surveillance System quality criteria in Chad.

Indicators	Expected score (%)	Observed score (%)	Assessment (Varkevisser)
Simplicity	100	72	Acceptable
Flexibility	100	39	Low
Acceptability	100	87	Good
Sensitivity	100	29	Low
PPV	100	29	Low
Reactivity	100	55	Acceptable
Representativeness	100	27	Low

**Quality of the IESS**	**100**	**44**	**Low**

IESS, Integrated Epidemiological Surveillance System; PPV, Positive Predictive Value.

## Appendix 7: Detailed Recommendations

**TABLE 1-A7:** Distribution of recommendations by levels of responsibility, identified issues and targets.

Level of responsibility	Problem identified	Recommendations	Targets
Central level	The dependence of Chad’s IESS on foreign countries (Cameroon and Senegal) in terms of confirmation of AFP cases.	Strengthen the capacity of the ES central laboratory to confirm AFP cases at the national level.	MPHP and WHO
	Low sensitivity and PPV of the system at home and abroad.	Strengthen the capacity of Heads of the Epidemiological Surveillance Units (HESU), FPs and clinicians on case definition (diagnostics) and procedures for the collection and safe transport of samples, both domestically and internationally.	MPHP and WHO
	Low representativeness of the IESS.	Consider the characteristics of representativeness (time, place, sex, age) in the IDSR database.	MPHP and WHO
	Unavailability of full response kits at the operational level.	Pre-prepare comprehensive disease response kits under surveillance in all health facilities.	MPHP and WHO
	Need for additional staff (SRN and HT) in the context of the ES.	Increase the number of FPs and community relays in the districts.	MPHP and WHO
	Challenges in obtaining financial information related to the ES at all levels.	To make available financial information related to the activities of the ES for the purpose of scientific research.	IES, PEV and COUSP Directorate
	Need for capacity building in ES expressed as a priority by FPs and clinicians in the Borkou and Logone Occidental regions	Train new surveillance FPs in SMIR and organise orientation sessions for HC (heath center) clinicians (hospitals and HCM) in all provinces, particularly in Borkou and Logone Occidental.	IES and WHO leadership
	Low availability of procedures (case definition) and updated operational guides for monitoring	Make available to Branch Heads and FPs all procedures, updated operational guides and management tools for all diseases under surveillance.	IESD
	Low feedback on data transmission and laboratory results	Produce monthly feedback of laboratory data and results, in the form of bulletins at all levels.	IES and WHO leadership
	Weak monitoring and evaluation of surveillance activities with Health Delegates, District Medical Officers, Heads of Branches and FPs	Hold regular biannual monitoring and evaluation meetings (review meeting) with Health Delegates, District Medical Officers, Branch Heads and FPs	IES and WHO Directorate
	Limited financial resources, means of communication and means of travel to regional and district surveillance services.	Mobilise and allocate financial resources, communications and travel to provincial and district surveillance services	MPHP and WHO
	Non-availability of TORs for HESU and focal points at the operational level	Replicate and disseminate TORs from SEAs and surveillance focal points at the operational level	IES and WHO Directorate
	Inadequacies of IAS Biological Reagents and Laboratory Consumables	Order reagents and consumables for the two IES sample analysis laboratories	MPHP and Partner (WHO)
	Lack of IT hardware for data management at different levels	Equip CASEs, focal points and laboratory technicians with computer tools (computers, printers, photocopiers, etc.)	MPHP and Partner (WHO)
	Non-payment or delay in the payment of fuel costs and allowances for 2024, HESU, focal points and technicians at the central level	Pay the 2024 (or earlier) fuel costs and allowances of all stakeholders involved in surveillance activities	MPHP and Partner (WHO)
	Lack of a picture box to support community actors’ monitoring activities	To develop and disseminate image boxes to support awareness-raising activities at the community level	MPHP and Partner (WHO)
Intermediate and peripheral level	Low frequency of quality formative supervision visits of district focal points	Regularly conduct quality training supervision visits of the SD focal points by applying the supervision grid in force	PHD/HD
	Weak capacity to analyse surveillance data for intermediate and operational level decision-making	Recruit a data analytics specialist (statisticians, biostatisticians, and/or epidemiologists) to analyse surveillance data in real time for timely and informed decision-making at the intermediate and operational levels.	PHD/HD
	Deficiencies in the archiving of data and investigation forms	Improve the physical and electronic archiving (servers or secure cloud) of records and databases	PHD/CASE and HD/PF
	Weak involvement of private and community structures in active surveillance activities (clinics, doctors’ and care practices, traditional practitioners)	Regularly update the list of priority sites taking into account private actors and strengthening community-based surveillance by involving key informants. Promote public-private partnership in the field of IES.	PHD/CASE and HD/PF
	Lack of an action plan integrating epidemiological surveillance at the operational level	Develop and implement action plans integrating surveillance at the provincial and HD levels	PHD/HC
	Failure to comply with health measures by the community during outbreaks.	Conduct awareness campaigns for the community about compliance with the preventive measures taken by the health authorities in the event of an epidemic.	MPHP and WHO
	Low confirmation rate of cases at the provincial level.	Equip provincial hospitals with the laboratory kits, in quantity and quality, necessary for the confirmation of cases at the provincial level.	MPHP and WHO
	Low vaccination coverage	Strengthen SIA campaigns and routine EPI by focusing on the ACD approach and the fixed, advanced and mobile strategy, or even door-to-door	PHDs, HCs and partners (WHO, UNICEF, etc.)

ACD, active case detection; AFP, Acute Flaccid Paralysis; COUSP, Centre des opérations d’urgences en santé publique; COVID, coronavirus disease 2019; EPI, Expanded Program on Immunisation; ES, Epidemiology Surveillance; ESS, Epidemiological Surveillance System; FP, focal point; HC, heath centre; HCM, Health Centre Managers; HD, heath district; HESU, Head of the Epidemiological Surveillance Unit; HT, healthcare technician; IDS, Intrusion Detection System; IDSR, Integrated Disease Surveillance and Response; IES, Integrated Surveillance System; IESS, Integrated Epidemiological Surveillance System; MPHP, Ministry of Public Health and Prevention; PHD, Public Heath Directorate; PPV, Positive Predictive Value; SIA, upplementary immunisation activities; SRN, State-Registered Nurse; TOR, terms of references; UNDP, United Nation of Development Program; UNICEF, United Nations International Children’s Emergency Fund; WHO, World Health Organisation.
